# Supplementary material for: Clinical outcomes and associated factors among mechanically ventilated patients in adult intensive care unit at Jimma Medical Center, Southwest Ethiopia, 2024
Source: PLoS One. 2026 Apr 21;21(4):e0341812. doi: 10.1371/journal.pone.0341812 (PMC13098938; doi:10.1371/journal.pone.0341812)
Supplement: Attachment — Submitted filename: Response_to_Reviewers_PLOS_ONE Edited.docx [file pone.0341812.s002.docx]

**Response to Reviewers**

| Reviewer / Editor | Comment No. | Reviewer Comment | Section & Line Number (Revised Manuscript) | Revision Made (Summary) | Author Response |
| --- | --- | --- | --- | --- | --- |
| Editor | 2 | Clarify participant consent, consent type, waiver, and anonymization. | Methods – Ethical Considerations (Lines 312–324) | Expanded ethics statement to specify retrospective chart review, full anonymization prior to data access, and IRB-approved waiver of informed consent. | We revised the Ethics section to clearly describe consent waiver and data anonymization, in accordance with PLOS ONE requirements. |
| Editor | 3 | ORCID iD required for corresponding author. | Editorial Manager (Administrative) ORCID iD: 0009-0002-0511-8625 | ORCID iD created and validated for corresponding author which is ORCID iD: 0009-0002-0511-8625 | The corresponding author has obtained and validated an ORCID iD in Editorial Manager. |
| Editor | 4 | Competing Interests statement must confirm adherence to PLOS ONE policies. | Competing Interests (Lines 346–349) | Added required statement confirming adherence to PLOS ONE data and materials sharing policies. | The Competing Interests section has been updated exactly as requested. |
| Editor | 5 | Provide a complete Data Availability Statement. | Data Availability (Lines 351–354) | Inserted complete Data Availability Statement indicating data are included in the manuscript and supporting files. | A complete Data Availability Statement has now been included. |
| Editor | 6 | Refer to Figures 1 and 2 in the text. | Results (Lines 388–410) | Added explicit in-text citations for Figures 1 and 2. | Figures 1 and 2 are now cited appropriately in the Results section. |
| Editor | 7 | Upload Figure 6 or remove references. | Results (Lines 430–438) | Removed Figure 6 and deleted all in-text references. | Figure 6 was removed as it did not add new information. |
| Editor | 8 | Tables 2 and 3 not cited in text. | Results (Lines 402–415) | Added in-text citations for Tables 2 and 3. | All tables are now properly referenced in the text. |
| Editor | 9 | Include captions for Supporting Information files. | Supporting Information (Lines 560–568) | Added captions and updated in-text citations. | Supporting Information files are now fully captioned |
| Reviewer #1 | 1 | Abstract incorrectly labels study as case-control. | Abstract (Lines 45–48) | Replaced 'retrospective cross sectional study' with 'retrospective chart review study'. | The study design has been corrected throughout the manuscript. |
| Reviewer #1 | 2 | Introduction redundant and recommendations misplaced. | Introduction (Lines 68–79); Discussion (Lines 520–548) | Removed redundant text and relocated recommendations to Discussion. | The Introduction was streamlined and recommendations moved appropriately. |
| Reviewer #1 | 3 | Clarify study design, missing data handling, VIF, ethics, and tense. | Methods (Lines 230–325) | Clarified retrospective design, reported missing data handling, specified VIF threshold, corrected ethical clarification, and revised tense. | All methodological concerns were addressed and clarified. |
| Reviewer #1 | 4 | Improper labeling of tables/figures and unclear confidence interval. | Results (Lines 486–487) | Corrected table/figure labels and clarified mortality CI to 95% CI: 49.4–58.6. | Results section revised for accuracy and clarity. |
| Reviewer #1 | 5 | Discussion lacks clarity and justification. | Discussion (Lines 535–575) | Rewrote discussion to include CI and strengthened evidence-based justification. | The Discussion has been substantially improved. |
| Reviewer #1 | 6 | References not formatted correctly. | References | Reformatted references according to PLOS ONE style. | References revised to comply with journal guidelines. |
| Reviewer #2 | Abstract | Inconsistent statistics, decimals, and generic recommendations | Abstract; Conclusion | Standardized statistical reporting and rewrote conclusions to be specific and evidence-based. | Abstract and Conclusion revised for consistency and clarity. |
| Reviewer #2 | Methods | Study area, population, variables, operational definitions unclear. | Methods (Lines 210–360) | Reorganized Methods, clarified study period, inclusion/exclusion criteria, variables, and operational definitions. | Methods section revised to improve clarity and alignment with objectives. |
| Reviewer #2 | Results | Poor synthesis, unclear figures, informal diagrams. | Results (Lines 380–500) | Improved synthesis, removed informal figures, clarified tables and outcomes. | Results section reorganized and clarified. |
| Reviewer #2 | Discussion | Comparisons unjustified; recommendations too broad. | Discussion (Lines 545–590) | Tailored discussion to objectives and resource-limited settings; removed unsupported comparisons | Discussion revised to strengthen scientific reasoning. |
